# Camptocormia as the presenting symptom in sporadic late onset nemaline myopathy: a case report

**DOI:** 10.1186/s12891-019-2942-0

**Published:** 2019-11-20

**Authors:** Matthias Türk, Armin M. Nagel, Frank Roemer, Ursula Schlötzer-Schrehardt, Christian T. Thiel, Martin Winterholler, Rolf Schröder

**Affiliations:** 1 Department of Neurology, Friedrich-Alexander University Erlangen-Nürnberg (FAU), Schwabachanlage 6, 91054 Erlangen, Germany; 2 Institute of Radiology, Friedrich-Alexander University Erlangen-Nürnberg (FAU), Maximiliansplatz 3, 91054 Erlangen, Germany; 3 Institute of Radiology, Friedrich-Alexander University Erlangen-Nürnberg (FAU), Maximiliansplatz 3, 91054 Erlangen, Germany; 4 Department of Ophthalmology, Friedrich-Alexander University Erlangen-Nürnberg (FAU), Schwabachanlage 6, 91054 Erlangen, Germany; 5 Institute of Human Genetics, Friedrich-Alexander University Erlangen-Nürnberg (FAU), Schwabachanlage 10, 91054 Erlangen, Germany; 6 Department of Neurology, Sana-Krankenhaus Rummelsberg, Rummelsberg 71, 90592 Schwarzenbruck, Germany; 7 Institute of Neuropathology, Friedrich-Alexander University Erlangen-Nürnberg (FAU), Schwabachanlage 6, 91054 Erlangen, Germany

**Keywords:** Camptocormia, Axial myopathy, Muscle biopsy, Nemaline rods, Sporadic late onset nemaline myopathy, SLONM

## Abstract

**Background:**

Camptocormia has been reported in a plethora of diseases comprising disorders of the central nervous system, the peripheral nervous system, and the neuromuscular junction as well as hereditary and acquired myopathies. In sporadic late onset nemaline myopathy concomitant axial myopathy is common, but reports about camptocormia as the only presenting symptom in this condition are very rare. Notably, sporadic late onset nemaline myopathy is a potentially treatable condition in particular when associated with monoclonal gammopathy of unknown significance, HIV or rheumatological disorders.

**Case presentation:**

We report the case of a 62-year-old female patient, who presented with slowly progressive camptocormia. Comprehensive work-up including neurological work-up, laboratory tests, MR-imaging, muscle biopsy and genetic testing led to the diagnosis of sporadic late onset nemaline myopathy.

**Conclusions:**

Our case report highlights that sporadic late onset nemaline myopathy has to be considered in patients presenting with isolated camptocormia and comprehensive work-up of camptocormia is mandatory to ascertain the individual diagnosis, especially in consideration of treatable conditions.

## Background

Camptocormia is characterized by involuntary forward flexion of the thoracolumbar spine. In contrast to spinal deformities due to skeletal disorders, forward flexion in this condition typically increases in upright and resolves in supine position [1]. Camptocormia has been reported in a plethora of diseases comprising disorders of the central nervous system (i.a. Parkinson’s disease, dystonias, psychiatric/psychogenic disorders), the peripheral nervous system (i.a. amyotrophic lateral sclerosis, chronic inflammatory demyelinating polyneuropathy), and the neuromuscular junction (myasthenia gravis) as well as hereditary (i.a. congenital myopathies, central-core-disease, mitochondrial myopathy, acid maltase deficiency, phosphorylase deficiency, fazio-scapulo-humeral muscle dystrophy, myotonic dystrophy type I/II, dysferlinopathy, calpainopathy, myofibrillar myopathies) and acquired myopathies (inflammatory myopathies (polymyositis; dermatomyositis; inclusion body myositis), amyloid myopathy, hypothyroid myopathy, toxic myopathy) [1–6]. In patients with primary myopathy, camptocormia is due to myogenic dysfunction and weakness affecting the thoracolumbar paraspinal/axial muscle groups. Whereas the manifestation of camptocormia is often preceded by weakness of extra-axial muscles, data on camptocormia as the only presenting symptom in primary myopathies and in particular in sporadic late onset nemaline myopathy (SLONM) is scarce [3, 7–10].

## Case presentation

**Fig. 1:**
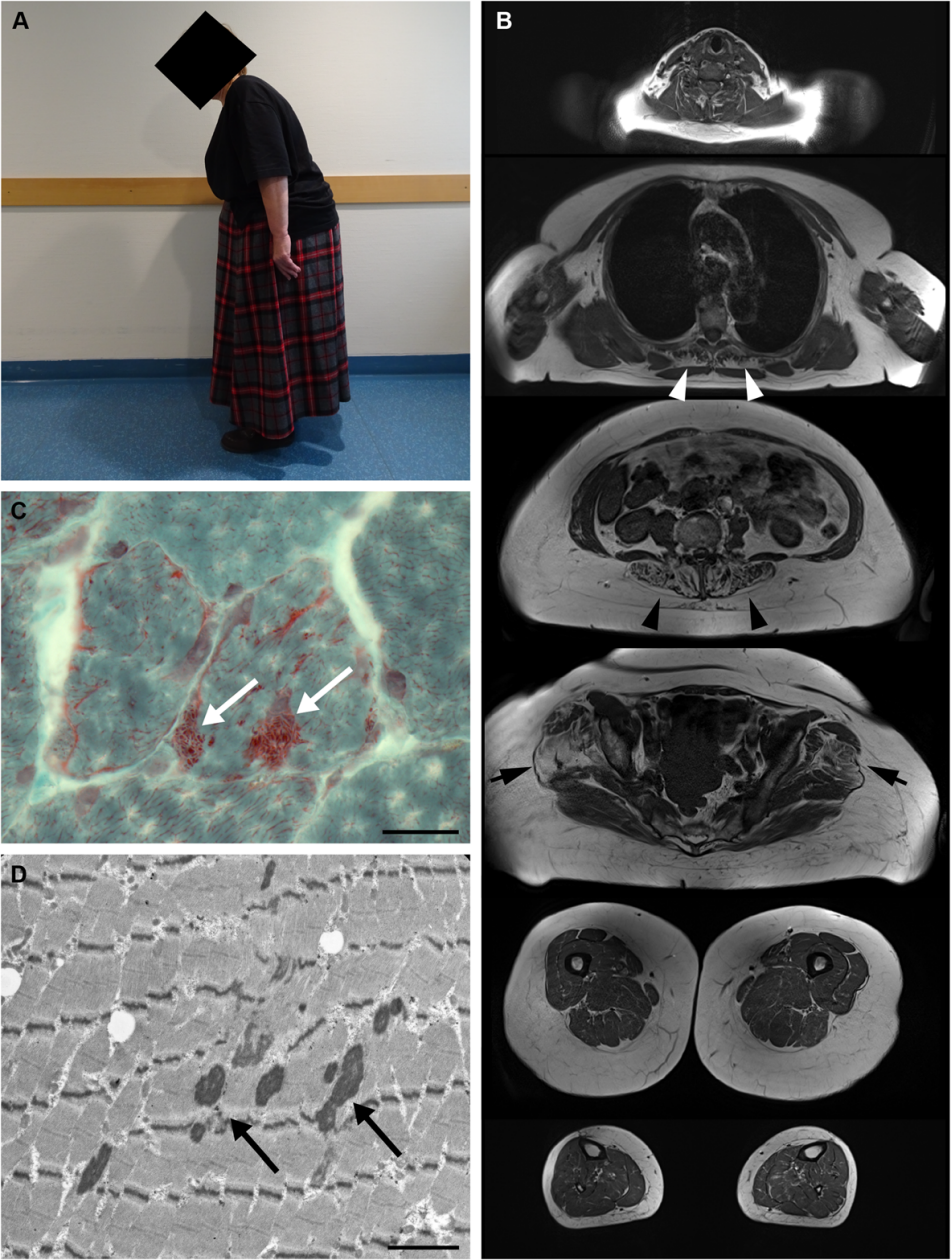
Clinical presentation, MR-imaging and muscle biopsy findings in the reported SLONM-patient. **a** Photograph depicts camptocormia while standing. **b** Representative transversal MR-images (T1 TSE tra) reveal diffuse and marked fatty atrophy of the thoracic (white arrowheads) and lumbar (black arrowheads) paraspinal musculature. Note moderate bilateral fatty atrophy of the antero-lateral part of the gluteus medius muscles and less marked of the gluteus minimus muscles (black arrows). **c** Histopathological analysis of Gomori trichrome stained section shows muscle fibers with multiple subsarcolemmal and sarcoplasmic nemaline rods (white arrows). Scale bar: 20 μm. **d** Electron microscopy detects multiple sarcoplasmatic electron-dense nemaline rods (black arrows). Scale bar: 1,2 μm

We report the case of a 62-year-old female patient of European origin, who presented with slowly progressive camptocormia. Her progressive inability to walk upright started at the age of 52 years and was accompanied by generalized myalgias, which were independent of exertion. Her family history was negative for neurological and neuromuscular disorders and neurological examination at age 60 revealed isolated axial muscle weakness resulting in camptocormia (Fig. 1a). During 2 years follow-up, camptocormia slightly progressed and very mild facial weakness and mild weakness of pelvic-girdle muscles developed. There were no signs of movement disorder, myasthenia or cardiopulmonary involvement. Laboratory tests, including standard parameters, creatine kinase (70 U/l), thyroid-stimulating hormone, routine rheumatologic tests, protein electrophoresis and immunofixation of serum and urine were within normal limits. HIV-testing was negative. Needle electromyography of the right vastus lateralis muscle showed some muscle unit action potentials with increased amplitudes, but was otherwise normal. Whole body 3 T-MR-imaging revealed marked fatty atrophy of the lumbar and thoracic paraspinal muscles in T1-weighted sequences (Fig. 1b) without signs of active myositis in STIR-sequences (data not shown). Apart from a moderate bilateral fatty atrophy of the antero-lateral part of the gluteus medius muscles and less marked of the gluteus minimus muscles, MR-imaging of shoulder- and pelvic-girdle- as well as arm- and leg-muscles was regular (Fig. 1b). Light and electron microscopy analysis of a diagnostic muscle biopsy from the left vastus lateralis muscle showed a myopathic pattern with nemaline rods (Fig. 1c/d) in about 4% of the muscle fibers along with some lobulated fibers. In addition, type-I- and type-II-fiber-grouping consistent with a mild chronic neurogenic pattern was noted. Next-generation sequencing did not show any mutations in genes associated with hereditary nemaline myopathy (α-tropomyosin 3; Nebulin; α-Actin; β-tropomyosin; Troponin T1; KBTBD13; Cofilin-2; KLHL40; KLHL41; LMOD3; MYPN; Ryanodine receptor 1) or any other myopathy-associated genes.

## Discussion and conclusions

Camptocormia as the presenting symptom has been reported in a wide variety of primary myopathies including inflammatory myopathies, amyloid myopathy, myotonic dystrophy, muscular dystrophies (FSHD1; dysferlinopathy, symptomatic carrier of dystrophinopathy, calpainopathy), metabolic myopathies (McArdle; Pompe’s disease), mitochondrial myopathies and toxic myopathy [3, 4].

In the reported patient with initially isolated camptocormia, our diagnostic work-up led to the diagnosis of sporadic late onset nemaline myopathy, which is defined as a late-onset and subacutely evolving muscle disorder. Schnitzler et al. recently published a systemic overview comprising a large cohort of 76 patients with SLONM, excluding patients with HIV infection [11]. In this analysis, the most typical clinical manifestations of SLONM were weakness and atrophy of proximal upper limb muscle groups followed by proximal lower limbs weakness. Axial weakness was reported in 68% of these patients. Less frequent symptoms were facial weakness, dyspnea, dysphagia, distal weakness, ophthalmoparesis, ptosis, myalgias and cramps. A very recent monocentric and retrospective case series by Naddaf et al. comprising 28 SLONM-patients reported axial weakness (i.e. head drop, camptocormia or general core weakness) as first noted symptom and as predominantly finding at presentation in 39 and 36% of all analyzed patients, respectively. In 47% of the reported patients, camptocormia was one of the presenting symptoms [12]. While axial weakness and camptocormia is a common feature, camptocormia as the only presenting symptom has been reported just in singular SLONM-patients [3, 8–10].

Though SLONM is rare, it is potentially a treatable condition. Patients with SLONM associated with HIV-infection or rheumatological diseases often show good clinical response to immunosuppressive therapy [11]. In SLONM patients without HIV-infection or rheumatological diseases therapy is still a matter of debate. Until recently, therapeutic interventions mainly focused on SLONM-patients with MGUS since those were assumed to have a more rapid progression and a very poor prognosis mainly due to respiratory failure [11, 13]. MGUS was detected in about half of the affected patients, analyzed in the study by Schnitzler et al. [11] In this subgroup of SLONM-patients, symptoms improved in several cases after immunosuppressive or -modulating therapy and especially after therapy with high-dose melphalan followed by autologous peripheral blood stem cell transplantation [11, 14, 15]. Interestingly, Naddaf et al. reported no difference in overall survival (92% in 5 years; 68% in 10 years) between SLONM-patients with or without MGUS. Notably, in their cohort all but one SLONM-patient (without MGUS) received immunotherapy. Based on their data, they recommend treatment with intravenous immunoglobulins as first line therapy in SLONM-patients irrespective of the presence of MGUS [12].

Regular check-ups including physical examination with special attention to cardiac and pulmonary testing are mandatory in SLONM-patients. Diagnostic work-up must repetitively address MGUS, HIV-infection [16] and rheumatological disorders [17, 18]. All of which were excluded in our patient by repeated laboratory testing and therapy was originally restricted to supportive care comprising physiotherapy and the use of nordic walking poles. However, treatment with intravenous immunoglobulins is reconsidered in our patient.

Whole body muscle MRI is a reliable diagnostic tool for detecting myopathy in patients with camptocormia and might further help to choose the site for muscle biopsy [6]. In our case, clinical diagnosis of camptocormia due to myopathy was supported by the MR-imaging findings which revealed a marked fatty atrophy almost exclusively affecting the lumbar and thoracic paraspinal muscles. Nevertheless, histopathological findings of nemaline myopathy were shown in a muscle biopsy from the vastus lateralis muscle. In this context, choosing the suitable site for biopsy in isolated axial myopathy is still a matter of debate, since normative findings for paraspinal muscle biopsy are lacking [5]. Noteworthy, deltoid muscle biopsy was shown to be able to detect an underlying myopathy in 35% of patients with isolated camptocormia [19] and apart from paraspinal muscles deltoid or vastus muscles might therefore be a possible site for muscle biopsy in these patients.

Our case report highlights that myopathy and in particular SLONM has to be considered in all patients with isolated camptocormia. SLONM should especially be suspected when camptocormica is combined with MGUS, HIV or rheumatological disease [12]. In all patients with camptocormia of unknown etiology, comprehensive work-up including muscle MRI and muscle biopsy is mandatory to ascertain the individual diagnosis, especially in consideration of treatable conditions.

## Data Availability

Data are contained within the manuscript.

## Abbreviations

HIV: Human immunodeficiency virus
MGUS: Monoclonal gammopathy of undetermined significance
SLONM: Sporadic late onset nemaline myopathy
